# Supplementary material for: New Caledonian crows keep ‘valuable’ hooked tools safer than basic non-hooked tools
Source: eLife. 2021 Dec 21;10:e64829. doi: 10.7554/eLife.64829 (PMC8691834; doi:10.7554/eLife.64829)
Supplement: Supplementary file 1. [file elife-64829-supp1.docx]

**SUPPLEMENTARY MATERIAL**

**New Caledonian crows keep ‘valuable’ hooked tools safer than basic non-hooked tools**

**Barbara C. Klump, James J. H. St Clair, and Christian Rutz**

**Supplementary file 1a.** New Caledonian crows trapped in 2012, 2013 and 2015. Given are alpha-numerical ring-code (bird ID), sex (F, female; M, male; based on morphology unless genetic sexing was available, *n* = 3), and approximate age (tentatively classified according to gape colouration). Crosses (X) indicate in which year(s) birds were trapped. For experimental treatments, X indicates that a subject was tested in the corresponding treatment, and (X) that it was tested, but did not contribute data to analyses (for further details, see ‘Materials and methods, Study site and subjects’ and ‘Materials and methods, Video scoring and statistics’ in the main text).

| **bird ID** | **sex** | **age** | **gape** | **trapped in** | | | **1.A** | **1.B** | **2.A** | **2.B** | **2.C** | **2.D** |
| --- | --- | --- | --- | --- | --- | --- | --- | --- | --- | --- | --- | --- |
|  |  |  |  | **2012** | **2013** | **2015** |  |  |  |  |  |  |
| AM0 | F | juvenile | 0 | x |  |  |  |  |  |  |  |  |
| AM1 | F | immature | 60 | x^a^ |  | x | x |  |  |  |  |  |
| AM2 | M | immature | 75 |  |  | x | x |  |  |  |  |  |
| AM3 | M | juvenile | 5 | x |  |  |  |  |  |  |  |  |
| AM5 | F | adult | 95 |  |  | x |  |  |  |  |  |  |
| AM6 | F | juvenile | 5 |  |  | x |  |  |  |  |  |  |
| AM7 | M | adult | 90 | x |  |  |  |  | x | x | x | x |
| AM9 | M | immature | 30 |  |  | x |  |  |  |  |  |  |
| AN1 | F | immature | 25 | x |  |  |  | x | x | x | x | x |
| AN4 | F | immature | 50 |  |  | x |  |  |  |  |  |  |
| AN5 | F | juvenile | 30 |  | x |  |  |  |  |  |  |  |
| AN6 | F | immature – adult | 85 |  |  | x |  |  |  |  |  |  |
| AN7 | M | adult | 100 |  |  | x |  |  |  |  |  |  |
| AN8 | F | juvenile | 3 | x |  |  |  |  | x | x | x | x |
| AN9 | F | adult | 95 |  | x |  | x | x | (x) | x |  |  |
| AP0 | F | immature | 60 | x^b^ | x |  | x | x | x | x |  |  |
| AP4 | M | adult | 100 | x | x |  |  |  |  |  |  |  |
| AP5 | F | adult | 85 |  | x |  |  |  |  |  |  |  |
| AP6 | F | adult | 100 | x |  |  |  |  |  |  |  |  |
| AS8 | F | juvenile | 30 |  |  | x |  |  |  |  |  |  |
| CA3 | M | immature | 80 |  |  | x |  |  |  |  |  |  |
| CC9 | M | juvenile | 5 | x |  |  |  |  |  |  |  |  |
| CE0 | F | adult | 100 | x | x |  | x | x | x | x | x | x |
| CH1 | F | immature – adult | 80 |  |  | x |  |  |  |  |  |  |
| CK6 | M | adult | 100 |  | x | x | x |  |  |  |  |  |
| CN1 | M | immature | 50 |  |  | x |  |  |  |  |  |  |
| CN7 | F | juvenile | 5 |  |  | x | x |  |  |  |  |  |
| CP3 | F | juvenile | 15 | x |  |  |  |  |  |  |  |  |
| CR8 | F | immature | 75 | x |  |  |  | (x) | x | x | x | x |
| CS9 | F | juvenile | 3 | x | x |  |  | x | (x) | x |  |  |
| CU0 | F | adult | 99 |  |  | x | x |  |  |  |  |  |
| EA670180 | M | immature | 35 |  |  | x | x |  |  |  |  |  |
| EA670182 | M | adult | 100 |  | x | x | x |  |  |  |  |  |
| EA9 | M | immature | 80 |  |  | x |  |  |  |  |  |  |
| EJ6 | M | immature | 60 |  | x |  |  |  |  |  |  |  |
| EK1 | M | adult | 100 |  | x | x |  |  |  |  |  |  |
| EM0 | M | adult | 100 |  | x |  |  |  |  |  |  |  |
| EM3 | M | immature | 30 |  | x |  |  |  | x | (x) |  |  |
| EP9 | M | adult | 100 |  | x |  |  |  |  |  |  |  |
| ER1 | F | adult | 100 | x |  |  |  |  |  |  |  |  |
| ER4 | F | juvenile | 5 | x | x |  | x | x | (x) | x |  |  |
| ER5 | F | juvenile | 20 |  |  | x | (x)^c^ |  |  |  |  |  |
| ER7 | F | juvenile | 10-15 |  |  | x |  |  |  |  |  |  |
| ES1 | M | immature | 40 | x |  |  |  |  |  |  |  |  |
| ES7 | F | adult | 100 |  | x |  |  |  |  |  |  |  |
| EU7 | F | immature | 60 | x |  |  |  |  | x | x | x | x |
| EU8 | F | juvenile | <1 |  | x |  | x | x | (x) | (x) |  |  |
| EV0 | F | adult | 95 | x |  |  |  | x | x | x | x | x |
| EV3 | M | immature | 40 | x^d^ | x |  | x | x | x | x |  |  |
| EV6 | F | adult | 100 |  |  | x |  |  |  |  |  |  |
| EV7 | F | juvenile | 1 |  |  | x |  |  |  |  |  |  |
| EV8 | M | immature | 35 |  |  | x |  |  |  |  |  |  |
| HA0 | M | adult | 100 | x |  |  |  |  |  |  |  |  |
| HA2 | F | immature | 75 |  |  | x |  |  |  |  |  |  |
| HA5 | F | adult | 100 |  | x^e^ | x | x |  |  |  |  |  |
| HA7 | F | immature | 25 | x |  |  |  | x | x | x | x | x |
| HA8 | F | immature | 20 |  | x |  | x | x | x | x |  |  |
| HJ2 | M | adult | 90 | x |  |  |  |  |  |  |  |  |
| HJ3 | F | adult | 97 |  | x^f^ | x | x |  |  |  |  |  |
| HJ4 | F | juvenile | 5 |  | x |  |  |  |  |  |  |  |
| HJ6 | M | immature | 35 |  | x |  | x | x | (x) | x |  |  |
| HJ7 | M | immature | 70 |  | x |  |  |  |  |  |  |  |
| no ring | M | adult |  |  |  | x |  |  |  |  |  |  |
| VV3 | M | immature | 50 | x |  |  |  |  |  |  |  |  |

^a^trapped in 2012 as immature, 20% gape colouration

^b^trapped in 2012 as juvenile, 5% gape colouration

^c^engaged with the experimental task, but did not produce valid data (prey extracted by bill; hooked stick tool broken); since the bird did not contribute data to analyses, it is not included in Supplementary file 1b (ii)

^d^trapped in 2012 as juvenile, 5% gape colouration

^e^trapped in 2013 as adult, 95% gape colouration

^f^trapped in 2013 as immature, 30% gape colouration

**Supplementary file 1b.** Percentages of New Caledonian crows (i) that were trapped, and (ii) that contributed data to analyses for Experiment 1 and/or Experiment 2 (data pooled), split by age and sex. For further information, see Supplementary file 1a.

(i)

| **trapped (*n =* 64)** | **females (*n =* 37)** | **males (*n =* 27)** |
| --- | --- | --- |
| juvenile | *n =* 14 (21.9%) | *n =* 2 (3.1%) |
| immature^*^ | *n =* 11 (17.2%) | *n =* 14 (21.9%) |
| adult | *n =* 12 (18.8%) | *n =* 11 (17.2%) |

(ii)

| **contributed data (*n =* 26)** | **females (*n =* 18)** | **males (*n =* 8)** |
| --- | --- | --- |
| juvenile | *n =* 5 (19.2%) | *n =* 0 (0%) |
| immature^*^ | *n =* 7 (26.9%) | *n =* 5 (19.2%) |
| adult | *n =* 6 (23.1%) | *n =* 3 (11.5%) |

^*^birds that were aged as ‘immature – adult’ (*n =* 2) were considered immatures for the purpose of percentage calculations

**Supplementary file 1c.** Point estimates^*^ and lower and upper limits of 95% confidence intervals (CI) (all on the log-odds scale) for the ten models reported in the main text and Figure 3. All response and explanatory variables were binary, and scored as follows (naming of explanatory variables follows the arrow labels in Figures 1 and 3): ‘level of safekeeping’ (i.e., whether or not tools were kept safe); ‘mode of safekeeping’ (i.e., how tools were kept safe – specifically, whether or not they were stored in holes); ‘material – tool type – manufacture effort’ (non-hooked stick tools crow-sourced from leaf litter *vs.* hooked stick tools crow-manufactured from *Desmanthus virgatus*); ‘tool type’ (non-hooked stick tools researcher-made from *D. virgatus vs*. hooked stick tools researcher-made from *D. virgatus*); ‘tool type – manufacture effort’ (hooked stick tools crow-manufactured from *D. virgatus* *vs.* non-hooked stick tools researcher-made from *D. virgatus*); ‘material’ (non-hooked stick tools crow-sourced from leaf litter *vs.* non-hooked stick tools researcher-made from *D. virgatus*); ‘manufacture effort’ (hooked stick tools crow-manufactured from *D. virgatus* *vs.* hooked stick tools researcher-made from *D. virgatus*). The conditions refer to the four experimental treatments in Experiment 2 (2.A–2.D), as described in the main text and illustrated in Figure 1.

| **model** |  | **condition** | **lower limit**  **for 95% CI** | **point**  **estimate** | **upper limit**  **for 95% CI** |
| --- | --- | --- | --- | --- | --- |
| #1 | level of safekeeping ~ ‘material – tool type – manufacture effort’ + (1\|bait) + (1\|birdID) | Intercept (2.B) | 2.26 | 3.41 | 4.56 |
|  |  | 2.A | -2.23 | -1.20 | -0.17 |
| #2 | mode of safekeeping ~ ‘material – tool type – manufacture effort’ + (1\|bait) + (1\|birdID) | Intercept (2.B) | -1.31 | 0.30 | 1.90 |
|  |  | 2.A | -2.50 | -1.71 | -0.91 |
| #3 | level of safekeeping ~ ‘tool type’ + (1\|bait) + (1\|birdID) | Intercept (2.D) | 2.05 | 3.60 | 5.15 |
|  |  | 2.C | -2.01 | -0.69 | 0.62 |
| #4 | mode of safekeeping ~ ‘tool type’ + (1\|bait) + (1\|birdID) | Intercept (2.D) | -2.01 | 0.28 | 2.56 |
|  |  | 2.C | -2.58 | -1.48 | -0.37 |
| #5 | level of safekeeping ~ ‘tool type – manufacture effort’ + (1\|bait) + (1\|birdID) | Intercept (2.B) | 2.23 | 2.90 | 3.57 |
|  |  | 2.C | -1.69 | -0.55 | 0.58 |
| #6 | mode of safekeeping ~ ‘tool type – manufacture effort’ + (1\|bait) + (1\|birdID) | Intercept (2.B) | -1.47 | 0.37 | 2.22 |
|  |  | 2.C | -2.12 | -1.08 | -0.04 |
| #7 | level of safekeeping ~ ‘material’ + (1\|bait) + (1\|birdID) | Intercept (2.C) | 1.33 | 2.86 | 4.39 |
|  |  | 2.A | -1.83 | -0.59 | 0.64 |
| #8 | mode of safekeeping ~ ‘material’ + (1\|bait) + (1\|birdID) | Intercept (2.C) | -2.82 | -0.73 | 1.35 |
|  |  | 2.A | -1.14 | -0.14 | 0.87 |
| #9 | level of safekeeping ~ ‘manufacture effort’ + (1\|bait) + (1\|birdID) | Intercept (2.B) | 2.12 | 3.35 | 4.58 |
|  |  | 2.D | -0.98 | 0.14 | 1.25 |
| #10 | mode of safekeeping ~ ‘manufacture effort’ + (1\|bait) + (1\|birdID) | Intercept (2.B) | -1.43 | 0.46 | 2.35 |
|  |  | 2.D | -0.65 | 0.07 | 0.78 |

^*^Point estimates of the intercepts are the log-odds of getting a 1 in the response variable when the tool is a hooked stick tool crow-manufactured from *D. virgatus* (2.B), a non-hooked stick tool researcher-made from *D. virgatus* (2.C), or a hooked stick tool researcher-made from *D. virgatus* (2.D). The second point estimate describes the change in log-odds (to get a 1 in the response variable) from the point estimate of the intercepts.
