# Supplementary material for: New Caledonian crows keep ‘valuable’ hooked tools safer than basic non-hooked tools
Source: eLife. 2021 Dec 21;10:e64829. doi: 10.7554/eLife.64829 (PMC8691834; doi:10.7554/eLife.64829)
Supplement: Transparent reporting form [file elife-64829-transrepform1.pdf]

eLIFE
1st Floor
24 Hills Road
Cambridge CB2 1JP, UK
P 01223 855340
W elifesciences.org
T @elife

eLife's transparent reporting form

We encourage authors to provide detailed information *within their submission* to facilitate the interpretation and replication of experiments. Authors can upload supporting documentation to indicate the use of appropriate reporting guidelines for health-related research (see EQUATOR Network), life science research (see the BioSharing Information Resource), or the ARRIVE guidelines for reporting work involving animal research. Where applicable, authors should refer to any relevant reporting standards documents in this form.

If you have any questions, please consult our Journal Policies and/or contact us: editorial@elifesciences.org.

Sample-size estimation

- You should state whether an appropriate sample size was computed when the study was being designed
- You should state the statistical method of sample size computation and any required assumptions
- If no explicit power analysis was used, you should describe how you decided what sample (replicate) size (number) to use

Please outline where this information can be found within the submission (e.g., sections or figure legends), or explain why this information doesn't apply to your submission:

In the section 'Materials and methods, Study site and subjects', we explain how we obtained our final sample of test subjects. Following recommendations of the STRANGE framework for animal behaviour research, we also report subject attribute and participation data (*Supplementary file 1a*) and included statements in the sections 'Materials and Methods, Study site and subjects' and 'Concluding remarks' describing potential sampling biases and limitations to the generalisability of our findings.

Replicates

- You should report how often each experiment was performed
- You should include a definition of biological versus technical replication
- The data obtained should be provided and sufficient information should be provided to indicate the number of independent biological and/or technical replicates
- If you encountered any outliers, you should describe how these were handled
- Criteria for exclusion/inclusion of data should be clearly stated
- High-throughput sequence data should be uploaded before submission, with a private link for reviewers provided (these are available from both GEO and ArrayExpress)

Please outline where this information can be found within the submission (e.g., sections or figure legends), or explain why this information doesn't apply to your submission:

Details about how many subjects participated in which experiment and treatment are provided in the section 'Materials and methods, Study site and subjects' and in *Supplementary file 1a*. We used a suite of pre-established inclusion/exclusion criteria, as outlined in the sections 'Materials and methods, Study site and subjects' and 'Materials and methods, Video scoring and statistics'; no outliers were removed.

eLife Sciences Publications, Ltd is a limited liability non-profit non-stock corporation incorporated in the State of Delaware, USA, with company number 5030732, and is registered in the UK with company number FC030576 and branch number BR015634 at the address 1st Floor, 24 Hills Road, Cambridge CB2 1JP | August 2014

Statistical reporting

- Statistical analysis methods should be described and justified
- Raw data should be presented in figures whenever informative to do so (typically when N per group is less than 10)
- For each experiment, you should identify the statistical tests used, exact values of N, definitions of center, methods of multiple test correction, and dispersion and precision measures (e.g., mean, median, SD, SEM, confidence intervals; and, for the major substantive results, a measure of effect size (e.g., Pearson's r, Cohen's d)
- Report exact p-values wherever possible alongside the summary statistics and 95% confidence intervals. These should be reported for all key questions and not only when the p-value is less than 0.05.

Please outline where this information can be found within the submission (e.g., sections or figure legends), or explain why this information doesn't apply to your submission:

This information can be found in the sections 'Materials and methods, Video scoring and statistics' and 'Results', Figure 2, Figure 3, and *Supplementary file 1b*.

(For large datasets, or papers with a very large number of statistical tests, you may upload a single table file with tests, Ns, etc., with reference to sections in the manuscript.)

Group allocation

- Indicate how samples were allocated into experimental groups (in the case of clinical studies, please specify allocation to treatment method); if randomization was used, please also state if restricted randomization was applied
- Indicate if masking was used during group allocation, data collection and/or data analysis

Please outline where this information can be found within the submission (e.g., sections or figure legends), or explain why this information doesn't apply to your submission:

Since we used a within-subject experimental design, randomisation was not necessary to allocate subjects to treatment groups. That said, we used randomisation procedures: to determine the position of the hooked and 'anomalous' tools in Treatments 1.A and 1.B in Experiment 1 ('Materials and methods, Experimental set-up and procedures'); to determine the order of treatments within subjects for both experiments ('Materials and methods, Experimental set-up and procedures'); to allocate stems to Treatments 2.C and 2.D in Experiment 2 ('Materials and methods, Experimental set-up and procedures'); to allocate prey types to log holes in Experiment 2 ('Materials and methods, Experimental set-up and procedures'); and to determine the order in which videos of both experiments were coded ('Materials and methods, Video scoring and statistics').

Additional data files ("source data")

- We encourage you to upload relevant additional data files, such as numerical data that are represented as a graph in a figure, or as a summary table
- Where provided, these should be in the most useful format, and they can be uploaded as "Source data" files linked to a main figure or table
- Include model definition files including the full list of parameters used
- Include code used for data analysis (e.g., R, MatLab)
- Avoid stating that data files are "available upon request"

Please indicate the figures or tables for which source data files have been provided:

The source data file for Figure 3 has been uploaded; *Supplementary file 1a* provides the source data for *Supplementary file 1b*.
